# ‘Opt-out’ referrals after identifying pregnant smokers using exhaled air carbon monoxide: impact on engagement with smoking cessation support

**DOI:** 10.1136/tobaccocontrol-2015-052662

**Published:** 2016-05-25

**Authors:** Katarzyna A Campbell, Sue Cooper, Samantha J Fahy, Katharine Bowker, Jo Leonardi-Bee, Andy McEwen, Rachel Whitemore, Tim Coleman

**Affiliations:** 1 Division of Primary Care, School of Medicine, UK Centre for Tobacco and Alcohol Studies, University of Nottingham, Nottingham, UK; 2 Division of Epidemiology and Public Health, School of Medicine, UK Centre for Tobacco and Alcohol Studies, University of Nottingham, Nottingham, UK; 3 National Centre for Smoking Cessation and Training (NCSCT), London, UK

**Keywords:** Cessation, Priority/special populations, Health Services

## Abstract

**Background:**

In the UK, free smoking cessation support is available to pregnant women; only a minority accesses this. ‘Opt-out’ referrals to stop smoking services (SSS) are recommended by UK guidelines. These involve identifying pregnant smokers using exhaled carbon monoxide (CO) and referring them for support unless they object.

**Methods:**

To assess the impact of ‘opt-out’ referrals for pregnant smokers on SSS uptake and effectiveness, we conducted a ‘before–after’ service development evaluation. In the 6-month ‘before’ period, there was a routine ‘opt-in’ referral system for self-reported smokers at antenatal ‘booking’ appointments. In the 6-month ‘after’ period, additional ‘opt-out’ referrals were introduced at the 12-week ultrasound appointments; women with CO≥4 ppm were referred to, and outcome data were collected from, local SSS.

**Results:**

Approximately 2300 women attended antenatal care in each period. Before the implementation, 536 (23.4%) women reported smoking at ‘booking’ and 290 (12.7%) were referred to SSS. After the implementation, 524 (22.9%) women reported smoking at ‘booking’, an additional 156 smokers (6.8%) were identified via the ‘opt-out’ referrals and, in total, 421 (18.4%) were referred to SSS. Over twice as many women set a quit date with the SSS after ‘opt-out’ referrals were implemented (121 (5.3%, 95% CI 4.4% to 6.3%) compared to 57 (2.5%, 95% CI 1.9% to 3.2%) before implementation) and reported being abstinent 4 weeks later (93 (4.1%, 95% CI 3.3% to 4.9%) compared to 46 (2.0%, 1.5% to 2.7%) before implementation).

**Conclusions:**

In a hospital with an ‘opt-in’ referral system, adding CO screening with ‘opt-out’ referrals as women attended ultrasound examinations doubled the numbers of pregnant smokers setting quit dates and reporting smoking cessation.

## Introduction

Smoking in pregnancy increases the risks of miscarriage, stillbirth, prematurity, low birth weight, perinatal morbidity and mortality, sudden infant death, asthma,^1^ learning difficulties,^2^ obesity and diabetes.^3^ It is a substantial public health problem in high-income countries; for example, 26% of UK women smoke at some point in pregnancy;^4^ in Japan^5^ and Canada,^6^ the prevalence rate is around 10%. Less robust data are available from low-income countries, but the WHO anticipates an epidemic of smoking in pregnancy within these jurisdictions.^7^ Eliminating smoking in pregnancy should be a priority for healthcare systems internationally. Additionally, as children of smokers are more likely to start smoking themselves,^8^ ^9^ when pregnant women achieve permanent cessation, longer term benefits may occur as a consequence of lower smoking rates in their children as adults.

Pregnancy is a life event that strongly motivates women to stop smoking; approximately half of UK women who smoke attempt cessation after conception,^9^ and there are effective interventions that can help them.^10^ ^11^ Although a few countries systematically provide smoking cessation support for pregnant women, this has been offered in the UK via the National Health Service (NHS) Stop Smoking Services (SSS) since 2000.^12^ Despite SSS being free of charge and widely available, only around 12% of pregnant smokers access this support; however, nearly half of those who do report quitting for at least 4 weeks.^13^

The need to improve the uptake of cessation support has focused attention on methods for engaging with pregnant women. WHO^14^ recommends that all women's smoking status should be assessed early in pregnancy to ensure that all pregnant smokers receive prompt cessation support. Until recently, UK midwives asked women about smoking at their first (‘booking’) antenatal appointment at 8–12 weeks of gestation and referred those who requested support to the SSS (‘opt-in’ referrals). When using self-report, up to 25% of smokers fail to disclose their smoking;^15^ ^16^ however, identification of smokers at booking using exhaled carbon monoxide (CO) was only reported by 50% of SSS in the UK in 2010–2011.^17^ Opportunities to screen for smoking at subsequent pregnancy appointments, such as at the ultrasound dating scan, routinely performed at around 12 weeks of gestation, are also missed. The UK National Institute for Health and Care Excellence (NICE) recommends that exhaled CO screening should be used to identify smokers in early pregnancy, and those who do not specifically object should be referred for support; such referrals are known as ‘opt-out’ referrals.^18^ Evidence for the efficacy of this approach is limited to two studies: an observational audit of Scottish SSS, where the few SSS which were then using CO identification and ‘opt-out’ referrals were noted to have higher referral rates;^19^ and a ‘before–after’ study that monitored referrals to SSS from two UK hospitals.^20^ This latter study suggested that, after new referral processes began, referral numbers increased but women's smoking cessation did not; however, no formal statistical testing was used, hindering interpretation of findings.^20^ A large UK hospital Trust decided to integrate CO identification of smokers and ‘opt-out’ referrals at the ultrasound dating scan; our aim was to compare women's rates of referral for and engagement with SSS support and of smoking cessation before and after this change in practice.

## Methods

### Study design

**Figure 1:**
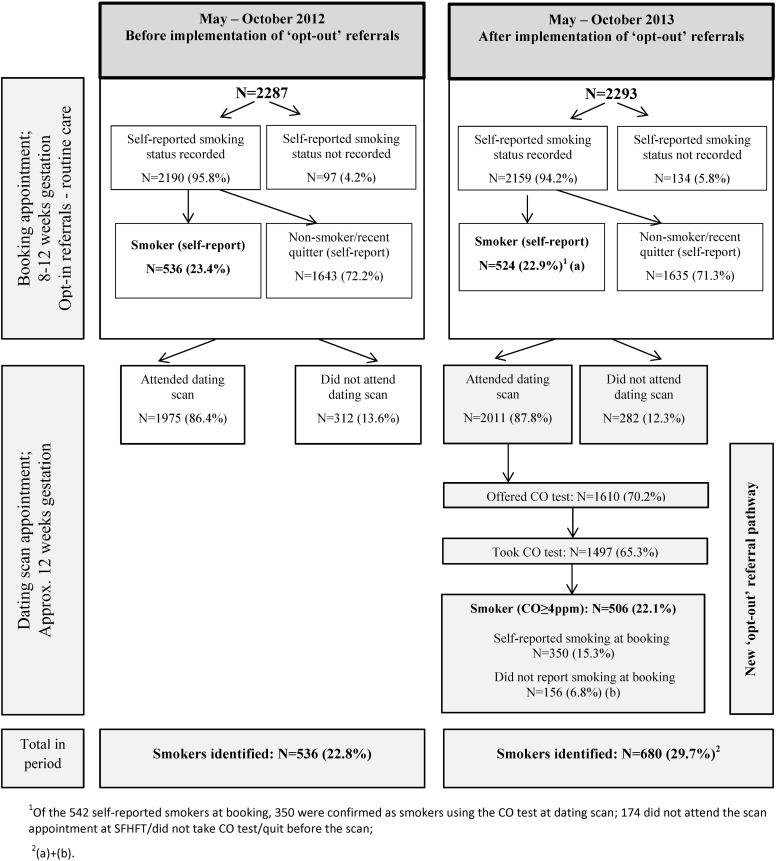
Patient flow and interventions at ‘booking’ and at dating scan appointments before and after implementation of the ‘opt-out’ referral pathway.

New ‘opt-out’ referral procedures with CO identification of smokers when attending their ultrasound dating scan were introduced at Sherwood Forest Hospitals NHS Foundation Trust (SFHFT), Nottinghamshire, UK between 1 May and 31 October 2013. SFHFT had two antenatal clinics: at Kings Mill Hospital (KMH) and Sherwood Women's Centre (SWC). For the study period, and for a comparison period between 1 May and 31 October 2012, anonymised data on referral processes and cessation outcomes were collected. Figure 1 shows patient flow and interventions delivered in both time periods.

This study is a ‘before–after’ evaluation of this service development.

#### May–October 2012: Routine practice before ‘opt-out’ referral implementation

Before the ‘opt-out’ referral pathway was introduced, there were already well-established procedures for referring pregnant smokers for cessation support within SFHFT. Routinely, at the first antenatal (‘booking’) appointment (8–12 weeks), midwives asked women about smoking and self-reported smokers who agreed were referred to SSS (‘opt-in’ referrals); CO monitors were not used. ‘Opt-in’ referrals were also offered at 25 and 34 weeks of gestation, at delivery and twice postnatally; however, few referrals were typically made at these points. Referrals were sent electronically to Nottinghamshire New Leaf, a community-based SSS, via a web-based system, integrated with electronic medical records (called Orion). Most women also attended two routine ultrasound appointments at SFHFT: a 12-week dating scan and a 20-week anomaly scan; smoking was not routinely discussed at these.

#### May–October 2013: Changes to practice after ‘opt-out’ referral implementation

**Figure 2:**
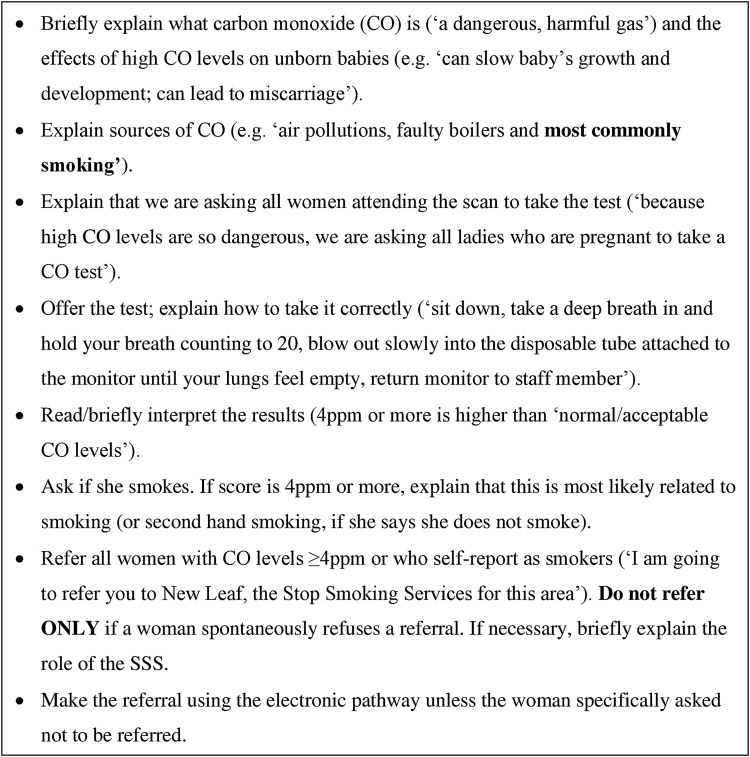
Protocol for ‘opt out’ referrals followed by healthcare assistants.

CO identification of smokers and ‘opt-out’ referrals were piloted in April 2013, and implemented, after routine ‘opt-in’ referrals at booking, between May and October 2013. This was implemented at the 12-week dating scan and was intended for all pregnant women who attended this appointment, regardless of their self-reported smoking status at booking. This time point was chosen as most pregnant women attend the 12-week scan, it is early in pregnancy and routine smoking cessation support was not offered at this point. We worked closely with SFHFT to integrate data collection into electronic medical records. The ‘opt-out’ referral pathway was implemented by five healthcare assistants at KMH and a midwife at SWC. All antenatal staff received a full-day training delivered by the National Centre for Smoking Cessation Training (NCSCT) based on evidence-based behaviour change techniques,^21^ and including risks of smoking in pregnancy; benefits of and challenges in smoking cessation; using CO monitors; interpreting CO readings; explaining risks associated with high CO; and making electronic referrals. Written materials reinforced oral presentations and group work. The protocol for delivering ‘opt-out’ referrals which healthcare assistants followed is described in figure 2. CO testing/referrals were prompted by the electronic medical record system. The pathway was integrated with routine observations, like blood pressure, which were already being made by these staff immediately after ultrasound examinations. The CO level ≥4 ppm was used to identify smokers based on NICE recommendations;^18^ past research^20^ found 4 ppm to be optimal in pregnancy. An additional healthcare assistant was appointed to work 20 hours/week for 6 months, managing any workload increase. An antenatal clinic manager acted as a local ‘champion’, overseeing the implementation and supporting the staff with new tasks. Similar to the ‘opt-in’ referrals, the ‘opt-out’ referrals were sent to SSS electronically via the Orion system. Staff were also monitored and supported by the research team.

### Smoking cessation support offered by SSS

Nottinghamshire New Leaf offered identical supportive interventions to all pregnant smokers referred in both periods. Staff attempted to call each woman twice, and if a woman was uncontactable, they sent a letter detailing ways to contact the SSS for support. Women who engaged with the service were encouraged to set a quit date and were offered weekly behavioural support for up to 12 weeks, and up to 12 weeks of nicotine replacement therapy in fortnightly batches on an abstinent–contingent basis at no-cost to them. The behavioural support offered to women was based on the NICE guidelines^18^ and NCSCT Standard Treatment Programme.^22^ After implementation, a role of ‘pregnancy lead’ was established within the service. She spent one half-day per week at the antenatal clinic, offering immediate support to pregnant smokers; this was discontinued due to the lack of demand.

### Data collection and participants

**Figure 3:**
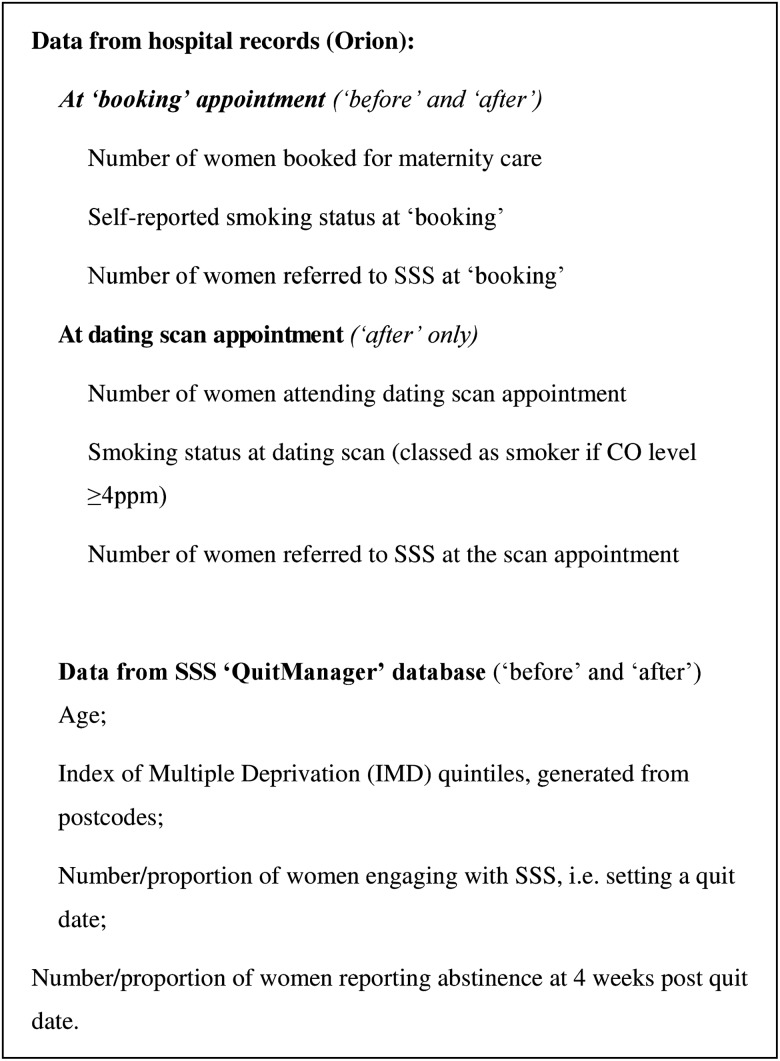
Data sources and data collected.

Anonymised, individual-level data were collected from two routine sources (figure 3). First, the Orion system provided ‘booking’ appointment data on the number of women receiving antenatal care and their smoking behaviour; this was also used for information about the ultrasound appointment. These data were obtained for all women presenting for antenatal care in each period. Second, from the SSS ‘QuitManager’ (North 51 HealthWare) database, we collected the available background information and outcome data. These data were collected for all pregnant women referred to the SSS from SFHFT via the ‘opt-in’ and/or ‘opt-out’ referrals.

### Study outcomes

After collection, data from both sources (figure 3) were cross-referenced to ensure accuracy and eliminate duplicate records.

#### Engagement

The primary study outcome and the one on which the study was powered (see below) was women's engagement with the SSS, defined as setting a quit date with SSS support, during pregnancy*.* Only women whom the SSS successfully contacted within 24 weeks after their referral were included in analyses, ensuring that women who engaged with SSS support after pregnancy were excluded. To ensure that only independent cases were analysed, individuals could only appear once in the data set and could only have one outcome. Only 10 women had more than one eligible record on QuitManager (1.4% of all referred). Where this occurred, we used the outcome of the latest record in pregnancy. As SSS offered pregnant smokers evidence-based support with cessation, engagement was considered an important outcome as greater engagement could be expected to result in more women stopping smoking.

#### Smoking cessation

At the time of the study in the UK, abstinence from smoking of an at least 2-week duration recorded at 4 weeks after starting a quit attempt was a mandatory outcome which the SSS reported to the English Department of Health; hence, these data were routinely available for all SSS clients.^23^ As study intervention was anticipated to affect women's engagement with the SSS and no substantial changes were made to the cessation support provided by the SSS, the 4-week post quit date cessation was considered an appropriate secondary outcome to measure the effectiveness of the ‘opt-out’ referral pathway.

### Data analysis

#### Sample size estimation

At the time of our a priori sample size estimation, we had access only to data from KMH, one of the two SFHFT hospitals; hence, our sample size estimate was conservative as the estimated throughput of smokers to SFHFT used was lower than the actual number of women attending antenatal care at both hospitals. As ∼800 women attended antenatal care at SWC annually, the actual number of women eligible for referral in each study period at both hospitals was around 800 higher than that in the sample size estimate below.

At KMH, in the 12 months before April 2012, 3286 women were booked; of those, 27.2% were smokers, 11.5% were referred to the local SSS and 3.0% set a quit date. Therefore, in each of the two study periods (ie, ‘before’ and ‘after’ intervention) anticipated to be 6 months long, at least 1684 pregnant women would be available for referral and a similar rate of ‘setting a quit date’ (ie, 3.0%) could be estimated with a 95% CI of 2.2% to 3.8% (±0.8% margin). We believed that a 1.5% absolute increase (ie, to 4.5%) in the proportion of pregnant women setting a quit date would be a positive outcome, and the study was powered such that this could be determined with a 95% CI of 0.137% to 0.163% (±0.13%).

#### Data analysis

Proportions of all women presenting for antenatal care who set a quit date and reported abstinence from smoking 4 weeks later are presented with 95% CIs, together with the difference in proportions between the study periods, again with 95% CIs. We report outcomes within pregnant women rather than within smokers because the method of identifying smokers varied in the two time periods and, hence, the population of pregnant women provided a denominator that was not prone to vary between periods. 95% CIs for single proportions were based on the Wilson method,^24^ and 95% CIs for the difference between two proportions were estimated based on the Newcombe-Wilson method without continuity correction.^25^ We also present descriptive statistics on the numbers of pregnant women receiving antenatal care and the proportions of smokers identified by usual Trust procedures at ‘booking’ for the two time periods. For the ‘after’ period, we present the number (percentages) of women offered/accepting CO tests and referred to SSS. Key characteristics of referred women are compared in study periods using χ^2^ and t-tests. As our analysis was based on routinely collected data, only information relating to age and socioeconomic status represented by the Index of Multiple Deprivations derived from postcode were of sufficient completeness for comparison. All analyses were carried out in Stata V.13.1 (StataCorp LP, USA).

## Results

### Identification of smokers at ‘booking’ and 12-week scan appointments

There were 2287 women booked for maternity care at SFHFT before and 2293 after implementation of the ‘opt-out’ referrals. As shown in figure 1, there were similar proportions of women who had their smoking status recorded and who reported smoking at ‘booking’ in both periods. Furthermore, similar proportions of women attended the scan at SFHFT in each period. Data on CO testing and ‘opt-out’ referrals at the 12-week dating scan are also presented in figure 1. As a result of the CO testing, 506 women (22.1%) were identified as smokers; of these, 350 had already reported being a smoker at ‘booking’, whereas a further 156, amounting to 22.9% of all smokers identified in this period, had not reported smoking at booking and were only identified as smokers via the CO testing. Overall, 536 individuals (22.8%) were identified as smokers in the period before and 680 (29.7%) in the period after implementation (524 at booking and an additional 156 at the dating scan).

### Outcomes of referrals received by SSS

Table 1 summarises that the proportion of all pregnant women who set a quit date with the SSS more than doubled in the period after implementation of the ‘opt-out’ referral pathway, equating to a significant increase of 2.8% (95% CI 1.7% to 3.9%). Furthermore, the proportion of all pregnant women who at 4 weeks after their quit date reported smoking cessation of an at least 2-week duration also doubled after implementation, equating to a significant overall increase of 2.1% (95% CI 1.1% to 3.1%).

**Table 1:** Referrals of pregnant smokers received by stop smoking services and outcomes before and after implementation of the ‘opt-out’ referral pathway

	Before: May–October 2012			After: May–October 2013			Difference in proportions (95% CI), (p value)
	N	Per cent	95% CI	N	Per cent	95% CI	
Women receiving antenatal care	2287			2293			NA
Referrals received by stop smoking services	290	12.7	11.4 to 14.1	421	18.4	16.8 to 20.0	5.7 (3.6 to 7.8)†
Women who set a quit date	57	2.5	1.9 to 3.2	121	5.3	4.4 to 6.3	2.8 (1.7 to 3.9)†
Women who reported stopping smoking 4 weeks after the quit date	46	2.0	1.5 to 2.7	93	4.1	3.3 to 4.9	2.1 (1.1 to 3.1)†

†p<0.001.

There was also a significant increase in the proportion of women referred to the SSS—12.7% (95% CI 11.4% to 14.1%) of women were referred in the period before versus 18.4% (95% CI 16.8% to 20%) after implementation of the ‘opt-out’ pathway. After implementation, 177 referrals were made at ‘booking’ and 323 during the dating scan appointments. Out of the 421 women referred after implementation, 98 women were referred at ‘booking’ only, 79 women were referred at both ‘booking’ and via the ‘opt-out’ pathway, and 244 women were referred via the ‘opt-out’ pathway only (data not in the table).

Furthermore, among women who were referred to the SSS, the quit rate before implementation was 15.9% (46 of 290) (95% CI 12.1% to 20.5%) compared to 22.1% (93 of 421) afterwards (95% CI 18.4% to 26.3%, p=0.04).

### Characteristics of women referred before and after introduction of the ‘opt-out’ pathway

Table 2 contrasts characteristics of the women who set a quit date in both time periods. These suggest that women setting a quit date in both periods were of similar age (t=0.1226, p=0.9026) and came from equally highly deprived areas (χ^2^=2.8263, p=0.587).

**Table 2:** Characteristics of smokers who set a quit date with stop smoking services before and after the implementation of the ‘opt-out’ referral pathway

Characteristic	Before (N=57)	After (N=121)	t-test/χ^2^ (p value)
Age	26.0	25.8	t=0.1226,
Median (IQR)	(22.7–30.3)	(21.9–30.2)	(p=0.9026)
IMD quintile, n (%)			
1 (least deprived)	2 (3.5)	1 (0.8)	χ^2^=2.8263 (p=0.587)
2	4 (7.0)	7 (5.8)	
3	10 (17.5)	15 (12.5)	
4	17 (29.8)	39 (32.5)	
5 (most deprived)	24 (42.1)	58 (48.3)*	

*Data on index of multiple deprivation (IMD) quintiles were not available for one of the women.

## Discussion

Introducing ‘opt-out’ referrals with CO identification of smokers at 12-week dating ultrasound scan appointments substantially increased the numbers of referrals for smoking cessation support received by the local SSS. Consequently, twice the number of women receiving antenatal care engaged with SSS support in the period after implementation. Crucially, there was also a doubling in the proportion of pregnant women who at 1 month after their quit date reported abstinence from smoking and an unexpected 6% statistically significant increase in successful cessation among women who used SSS in the intervention period.

The non-randomised study design means that there may be alternative explanations for our findings; we cannot, with absolute certainty, attribute these to the intervention. However, by using a time-matched comparison period, we have controlled for the potential effects of annual stop smoking campaigns, such as ‘Stoptober’.^26^ Furthermore, similar numbers of women received antenatal care before and after the intervention and similar patterns of smoking behaviours at the outset of maternity care were observed; so, it also seems unlikely that differences in smoking habits between the two periods could explain the findings. Also, SFHFT made no other changes to pregnant smokers' referral procedures and the local SSS did not implement alterations to their response to referrals. Finally, national data presented to the Department of Health by the SSS suggest that, in 2013, the proportion of pregnant smokers who set quit dates with SSS support fell for the second consecutive year across the UK, so the increase we observed was against the national trend.^13^ Therefore, it seems most likely that the new referral procedures were responsible for women's increased cessation activity.

Generalisability of findings is potentially an issue; referral procedures were introduced into an acute hospital trust serving a disadvantaged neighbourhood in the East Midlands, UK, where awareness of SSS is likely to be poor and smoking rates at the time of delivery are significantly higher than the national average (21% vs 12%, respectively, in 2013–2014).^27^ Although our findings may be most generalisable to maternity hospitals in areas with higher than average smoking rates, it is encouraging that effects observed were additional to those of existing, systematic ‘opt-in’ referral procedures. Furthermore, 22.9% of all smokers identified via the CO test failed to report smoking at booking; this phenomenon of pregnant smokers concealing their smoking status has been noted in other studies.^15^ ^16^ Introducing similar smoker-identification and referral methods into maternity hospitals with a less systematic routine care could result in even greater improvements in smoking cessation.

Being an evaluation set in a clinical care setting was a study strength that posed challenges; we used routine rather than specially collected data to monitor referral processes and smoking outcomes. Although our cessation outcome was not CO validated, methods used to collect these data had been long-established and formed the basis of SSS performance data submitted to the English Department of Health; although some smokers might have concealed their smoking at follow-up, there is no reason to suppose the prevalence of this potential bias would differ between study periods. We are confident that all women referred to SSS from this Trust were identified. Although we worked closely with hospital information technology staff to integrate the new referrals into the electronic hospital system, some referred women had no Orion record entry of a CO reading; however, it would be very unlikely for a referral to be made without CO monitoring. This suggests that the ‘opt-out’ referral pathway may have been implemented more comprehensively than we have observed, with CO monitoring being offered to more than 80% (1610 of 2011) of women attending ultrasound appointments who had this recorded in their medical records.

Only one other study has assessed the implementation of near-identical ‘opt-out’ referral processes.^20^ This study was set in two West Midlands maternity hospitals and used a ‘before–after’ design; the authors found an increase in pregnancy referral numbers, but not in cessation. Proportions setting quit dates were not reported. We are uncertain why similar referral procedures used in our study had such a positive impact; however, in our study, referral procedures were implemented more comprehensively (80% of women attending scan vs 61% in the West Midlands) and these were offered at ultrasound appointments rather than at ‘booking’.^20^ Seeing the baby for the first time, coupled with getting the CO reading and learning that high CO levels are harmful to the fetus, could have been an additional motivator for the women. At SFHFT, ‘opt-out’ referrals were implemented by a small group of healthcare staff, who were trained to national standards and received support afterwards; staff training and ongoing support may be necessary to ensure that new referral processes are effectively introduced. Finally, the ‘opt-out’ pathway was implemented in addition to existing ‘opt-in’ referrals, and repeated referrals may have enhanced smokers' motivation leading to improved cessation outcomes.

Other reports of ‘opt-out’ referrals are descriptive. In Glasgow, implementation across three antenatal units was inconsistent, with only 55% of pregnant women being offered a CO test overall; however, this figure masks variation between different hospitals.^28^ In one, where ancillary nurses conducted referrals, 89% of pregnant women provided CO samples, but in another where midwives oversaw the process only 35% did. The healthcare assistants who conducted referrals at SFHFT would be more similar to the now discontinued auxiliary nurse grade than midwives, suggesting that responsibility for overseeing these processes is best delegated to health professionals who routinely deal with the less complex aspects of patient care. Our findings indicate that with ongoing support and tailored training, support staff can effectively implement CO testing with ‘opt-out’ referrals and this process could be easily replicated in other antenatal hospitals.

In this study, there was a substantial increase in numbers of pregnant smokers identified and referred for further support; this may have additional, longer term effects, as SSS deliver evidence-based cessation support, which would be expected to encourage more women to achieve permanent abstinence from smoking.^10^ It could allow the SSS to target more women in the future to re-engage in treatment and prevent return to smoking after delivery. The additional knowledge imparted during the screening by trained antenatal staff may also have impacted on women's motivation to engage with SSS support and change general attitudes towards smoking in pregnancy.

Despite early systematic identification of pregnant smokers being recommended by UK national and international guidance,^14^ ^18^ antenatal staff are cautious about introducing CO testing with their patients.^29^ ^30^ By indicating that systematic implementation of this pathway can improve engagement and cessation outcomes in pregnancy, study findings could help reduce perceived barriers to introducing these procedures.

Implementation of ‘opt-out’ referrals with CO identification of smokers has some financial implications, such as staff and training costs or the cost of CO monitors. The increase in referral numbers may also affect SSS workload. While detailed economic evaluation would be necessary to fully investigate the cost-effectiveness of ‘opt-out’ referrals, the very positive cessation outcomes from this evaluation suggest that such further research is warranted as these methods could prove to be highly cost-effective.

## Conclusions

Systematic CO monitoring of all pregnant women with ‘opt-out’ referrals, introduced at the time of the first antenatal ultrasound appointment, has the potential to improve engagement of pregnant smokers with smoking cessation support and to improve cessation outcomes. This approach could be particularly advantageous in socially deprived areas, where prevalence of smoking during pregnancy is high and awareness of cessation services may be low.

**What this paper adds**

- We evaluated the impact of ‘opt-out’ referrals in pregnancy on stop smoking service (SSS) uptake and effectiveness within natural settings in one UK hospital Trust.
- Our evaluation indicated that ‘opt-out’ referrals with carbon monoxide screening delivered systematically by dedicated and trained healthcare assistants at the point of the first antenatal scan have the potential to significantly increase the uptake of cessation support in pregnancy and greatly improve cessation outcomes.
